# Functional pathway mapping analysis for hypoxia-inducible factors

**DOI:** 10.1186/1752-0509-5-S1-S3

**Published:** 2011-06-20

**Authors:** Chia-Sheng Chuang, Tun-Wen Pai, Chin-Hua Hu, Wen-Shyong Tzou, Margaret Dah-Tsyr Chang, Hao-Teng Chang, Chih-Chia Chen

**Affiliations:** 1 Department of Computer Science and Engineering & Center of Excellence for Marine Bioenvironment and Biotechnology, National Taiwan Ocean University, No. 2, Peining Road, Keelung 20224, Taiwan R.O.C; 2 Institute of Bioscience and Biotechnology, National Taiwan Ocean University, Keelung, Taiwan; 3 Institute of Molecular and Cellular Biology, National Tsing Hua University, Hsinchu, Taiwan; 4 Graduate Institute of Molecular Systems Biomedicine, China Medical University, Taichung, Taiwan

## Abstract

**Background:**

Hypoxia-inducible factors (HIFs) are transcription factors that play a crucial role in response to hypoxic stress in living organisms. The HIF pathway is activated by changes in cellular oxygen levels and has significant impacts on the regulation of gene expression patterns in cancer cells. Identifying functional conservation across species and discovering conserved regulatory motifs can facilitate the selection of reference species for empirical tests. This paper describes a cross-species functional pathway mapping strategy based on evidence of homologous relationships that employs matrix-based searching techniques for identifying transcription factor-binding sites on all retrieved HIF target genes.

**Results:**

HIF-related orthologous and paralogous genes were mapped onto the conserved pathways to indicate functional conservation across species. Quantitatively measured HIF pathways are depicted in order to illustrate the extent of functional conservation. The results show that in spite of the evolutionary process of speciation, distantly related species may exhibit functional conservation owing to conservative pathways. The novel terms OrthRate and ParaRate are proposed to quantitatively indicate the flexibility of a homologous pathway and reveal the alternative regulation of functional genes.

**Conclusion:**

The developed functional pathway mapping strategy provides a bioinformatics approach for constructing biological pathways by highlighting the homologous relationships between various model species. The mapped HIF pathways were quantitatively illustrated and evaluated by statistically analyzing their conserved transcription factor-binding elements.

**Keywords:**

hypoxia-inducible factor (HIF), hypoxia-response element (HRE), transcription factor (TF), transcription factor binding site (TFBS), KEGG (Kyoto Encyclopedia of Genes and Genomes), cross-species comparison, orthology, paralogy, functional pathway

## Background

It is a challenge for aerobic life to maintain oxygen homoeostasis due to environmental changes and energy demands. In all metazoans hypoxia-inducible factors (HIFs) are transcription factors (TFs) that play a central role in adaptive processes in hypoxic cellular environments [1,2]. HIF-1 is a heterodimeric protein composed of an oxygen-sensitive α-subunit (HIF-1α) and a ubiquitously expressed β-subunit (HIF-1β) also called aryl hydrocarbon receptor nuclear translocator (ARNT) [3]. HIF-1α contains 4 functional domains including a basic-helix-loop-helix (bHLH) domain for DNA binding [4,5], a PER-ARNT-SIM (PAS) domain for dimerization, an oxygen-dependent degradation (ODD) domain for targeting proteosomes, and transactivation domains (N-TAD and C-TAD) for transcriptional activation. HIF-1β contains bHLH, PAS, and TADs. Under hypoxic conditions, accumulated HIF-1α translocates from the cytoplasm to the nucleus and dimerizes with HIF-1β *via* the bHLH and PAS domains to form the HIF-1 complex. HIF-1α may recruit transcription co-activator P300/CBP and bind to a hypoxia response element (HRE) in the regulatory regions of hypoxia-inducible genes, thus mediating transcriptional activation. The consensus HRE motif is a *cis*-regulatory element with a core segment of 5′RCGTG3′ (where R is A or G) [6] that governs the transcription of HIF-responsive target genes in the hypoxia-signaling pathway such as those encoding proteins involved in oxygen transport, iron metabolism, glucose transport, cell proliferation, angiogenesis, invasion, and metastasis [7,8]. Interestingly, the overexpression of HIF-1α and activation of HIF pathways are observed in tumor cells due to lack of HIF-α ubiquitination and degradation in cancer patients. Hence, the inhibition of HIF-1 expression or activity is an alternative strategy in new cancer therapies [9].

A metabolic pathway represents a sequence of chemical reactions catalyzed by enzymes; most metabolic pathways retain the same functions in all growth stages of living cells. A signal transduction pathway starts with a signal to a receptor and ends with a change in cell behavior [10]. Hence, signal transduction pathways are implicated in different stages of dynamic transitions of a network in living cells. Both metabolic and signal transduction pathways are important components of physiology and are regulated by diverse mechanisms [11]. The HIF pathway is identified as a signal transduction pathway triggered due to low oxygen supply [12,13]. The classical representation of a biological pathway provides various associations among genes and proteins as well as system-level insight to discover functional information through molecular interaction. During the last decade, an increasing number of pathway datasets has been established in order to correlate functional interactions within a network and elucidate regulatory mechanisms. Hence, biological pathway analysis may facilitate the design and development of biological experiments.

Based on the evolutionary conservation of genomes of related organisms, the construction of a phylogeny, known as phylogenomics, can be carried out. Accordingly, the validity of phylogenetic analysis provides evolutionary relationships among species [14]. Furthermore, phylogenetic inferences can facilitate the understanding of species derivation and the delineation of homologous genes [15]. Random mutations accumulated over the course of many generations may evolve homologous genes that comprise 2 major categories: orthologous and paralogous genes. The former evolves directly from an ancestral gene through speciation events to daughter species, and the latter diverges after gene duplication events within a single species. Most orthologous genes retain similar functions during the course of evolution, while paralogous genes may gain new functions. The history of orthologous genes reflects the development track of diversified species [16]. Hence, functional divergence studies based on cross-species gene comparison may facilitate the prediction of protein functions and identification of horizontal gene transfer events [17]. Here, a methodology for mapping specific biological pathways across several model species and discovering conserved TF-binding motifs from related homologous genes is established for conserved pathway analysis. When a partial subset of the constructed pathway of the target species disappears, it is possible to efficiently replace the empty nodes by retrieving corresponding paralogous genes possessing similar biological functions [18].

To evaluate the importance of retrieved orthologous and paralogous genes within the mapped functional HIF pathways, a conventional strategy of searching conserved transcription factor binding sites (TFBSs) by matrix-based search on all retrieved HIF target genes was performed. For a selected homologous gene set, the TRANSFAC database containing a comprehensive set of TF-binding specificities summarized as position-specific scoring matrices (PSSMs) was adopted to identify conserved motifs for transcriptional activities [19,20]. All identified TFBSs from the retrieved homologous genes within a functional pathway among various species are statistically analyzed and ranked in priority order according to the total number of species possessing common motifs.

## Results

### Mapping HIF pathways among various species

**Figure 1:**
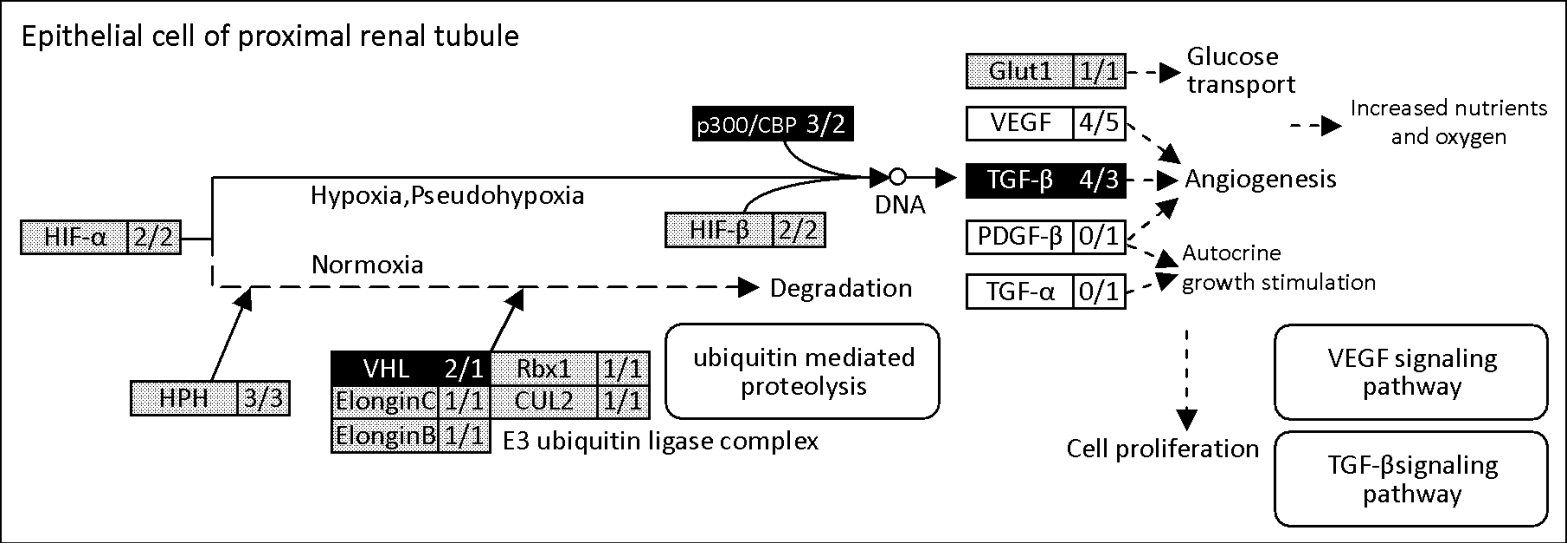
A mapped HIF functional pathway with zebrafish as the query species and humans as the reference species is shown. The initial HIF pathway was acquired from the KEGG database, and the mapped functional pathway between zebrafish and humans was constructed on the basis of the homologous relationship. The novel parameters OrthRate and ParaRate are proposed for indicating the degree of functional conservation across species regarding a specific biological function. Higher ParaRates are shown within rectangular boxes with darker backgrounds. The rectangular boxes with white background represent no orthologous relationship at the compared node between 2 species.

Based on the evidence of evolutionary conservation in genetic sequences and functions, it is reasonable to assume that the functional pathways of a given species could be mapped by cross-species inference. Orthologous relationships among various species and paralogous properties within an individual species were compared using public data for *Homo sapiens* (HSA), *Mus musculus* (MMU), *Gallus gallus* (GGA), *Danio rerio* (DRE), *Xenopus tropicalis* (XTR), and *Ciona intestinalis* (CIN). Orthologous relationships show the conservation of genes in a strong sense, which not only holds reservation in species evolution but also reflects the importance of functional pathways. Using human renal cell carcinoma (KEGG pathway: hsa05211) as an example while focusing on the HIF subpathway shown in Figure 1, a total of 14 genes are displayed in the rectangular boxes, which are correlated with the HIF transcriptional complex and respond to changes in cellular oxygen levels. It is also reported that when the HIF system is activated, HIF binds to the consensus HRE motifs present in the oxygen-regulated regions of HIF target genes such as Glut1, VEGF, TGF-β, PDGF-β, and TGF-α. The total number of orthologous and paralogous genes within HIF pathways in various species as well as the OrthRate and ParaRate parameters in comparison to *H. sapiens* were accessed and calculated. Table 1 shows that the number of orthologous genes (OrthNum) of HSA, MMU, GGA, and DRE possess almost equivalent numbers of genes in the HIF functional pathway. In contrast, both XTR and CIN apparently possess less orthologous genes than the other 4 species. This result implies that HSA, MMU, GGA, and DRE might possess more similar mechanisms to maintain HIF function than XTR and CIN. Hence, functional similarity between 2 species can be easily determined by the OrthRate index (defined in *Method*s) by using 1 species as the reference model species. Based on the high functional similarities shown in the mapped biological pathways and OrthRate analysis between 2 species, the comparison strategy provides a tip for suggesting suitable model organism candidates for subsequent *in vitro* or *in vivo* experimental designs. In addition, a mapped subpathway with genes maintaining biological functions was constructed according to the characteristics of paralogous gene distribution. A biological network with higher flexibility enhances survival rates when patients with cancers are receiving clinical drug therapy, which involves the activation of novel pathways to maintain normal physiological functions. Hence, the ParaRate (defined in *Methods*) is applied to manifest the flexibility and substitutional possibilities for target genes. From the first column in Table 1, the phylogenetic distance relationship shows that DRE possesses a longer evolutionary distance to HSA than XTR. Interestingly, DRE has a higher ParaRate compared to XTR, suggesting a stronger functional conservation of HIF pathways despite the evolutionary process of speciation. Quantitative evaluation of the OrthRate and ParaRate parameters with respect to HIF pathways among HSA and other model species are not thoroughly consistent with the phylogenetic relationships. Therefore, based on the assumptions of functional conservation for highly homologous genes across species, we suggest that a quick analysis of mapped functional pathways among various model species should be performed prior to biological experiments. Cross-species functional analysis provides a good starting point to assist model species selection and recommends accurate novel paralogous genes in a flexible and alternative route for further investigation.

**Table 1:** Statistics of the OrthRate and ParaRate parameters within mapped functional pathways between 2 model species with humans as the reference

	Phylogeny	OrthNum	OrthRate	ParaNum	ParaRate
HSA	1	14	N/A	24	N/A

MMU	2	14	14/14 (100%)	26	26/24 (108.3%)

GGA	3	13	13/14 (92.9%)	20	20/24 (83%)

XTR	4	9	9/14 (64.3%)	8	8/24 (33%)

DRE	5	12	12/14 (85.7%)	23	23/24 (95.8%)

CIN	6	9	9/14 (64.3%)	9	9/24 (37.5%)

The first column, Phylogeny, shows the evolutionary distance from HSA to the other 5 model species. OrthNum represents the total number of genes involved within the subpathway of HIF responses, and OrthRate represents the functional conservation of the HIF pathway between 2 species (with HSA as the reference species). ParaNum shows the total number of paralogous genes involved within the subpathway of HIF responses, and ParaRate represents the relative possibility of gene replacement within the HIF functional pathway between 2 species (with HSA as the reference species). HSA, *H. sapiens*; MMU, *M. musculus*; GGA, *G. gallus*; DRE, *D. rerio*; XTR, *X. tropicalis*; CIN, *C. intestinalis*.

### Identification of HRE motifs in HIF target genes

Based on the retrieved HIF pathway of HSA from KEGG (hsa05211), the HIF target genes (Glut1, VEGF, TGF-β, PDGF-β, and TGF-α) and their corresponding paralogous genes from other species were collected. According to previous reports, functional HRE motifs appearing within promoter regions of HIF target genes were analyzed [6], and HREs within homologous genes were identified using PSSM alignment. The exact core segment and bilateral tolerant segments of HREs were obtained and formulated as a novel matrix for motif searching and evaluation. The newly created HRE transcription factor was named “V$HIF_STKE_2005” and used for the identification of conserved TFBSs. The V$HIF_STKE_2005 HRE patterns found in homologous genes of vascular endothelial growth factor B (VEGFA_B, KEGG: Orthology K05448) from various species (HSA: 3, MMU: 3, GGA: 1, DRE: 1, and CIN: 1) are shown in Table 2. Only the top 3 matched HRE motifs containing exactly matched core segments with corresponding locations are displayed in detail. The results show that all the orthologous genes concerning the VEGF node in the HIF pathways contain the important TFBS for V$HIF_STKR_2005. Furthermore, all retrieved paralogous genes indeed possess HRE motifs; these paralogous genes might be considered as alternative HIF target genes for DNA binding within the HIF functional pathway. It should be noted that there might be many genes possessing similar HRE motif patterns. However, genes lacking orthologous or paralogous relationships were excluded in this study.

**Table 2:** Identification of HRE motifs for HIF orthologous target genes

Model Species	HIF orthologous target genes (KEGG: Orthology K05448)		
HSA	ENSG00000119630 GGCAGG**CGTG**CAGACTCA Loc:-963~-946 TGTGTC**CGTG**CCTGGCTA Loc:-1392~-1375 GCCCCT**CGTG**GGTGGGCA Loc:-628~-611	ENSG00000112715 TGAGGA**CGTG**TGTGTCTG Loc:-517~-500 TGCATA**CGTG**GGCTCCAA Loc:-982~-965 TGTGTG**CGTG**TGGGGTTG Loc:-485~-468	ENSG00000173511 CGAGAT**CGTG**CCCCGGGG Loc:-641~-624 GGAGCG**CGTG**TCTGGGTC Loc:-277~-260 CTCACG**CGTG**CCACGGAG Loc:-1601~-1584

MMU	ENSMUSG00000004791 TGAGCA**CGTG**TGGATCCT Loc:-542~-525 CCAATC**CGTG**TGTGCTCA Loc:-204~-187 ATGTCA**CGTG**AAATGACG Loc:-122~-105	ENSMUSG00000023951 TGCATA**CGTG**GGTTTCCA Loc:-1082~-1065 AGTCTG**CGTG**AGGGAGGA Loc:-1538~-1521 TGAGTG**CGTG**CATGCATG Loc:-1570~-1553	ENSMUSG00000024962 TCCCCT**CGTG**AGGCAGCG Loc:-1799~-1782 ACTACA**CGTG**CAATAAAC Loc:-1726~-1709 GTCAAG**CGTG**CTGAGGCC Loc:-287~-270

GGA	ENSGALG00000010290 CCCCGA**CGTG**CGGAGCGG Loc:-1970~-1953 TGGCAC**CGTG**CTGGAATA Loc:-143~-126 CCCCAT**CGTG**CAGCCCCA Loc:-208~-191	N/A	N/A

DRE	ENSDARG00000034700 CCTGTA**CGTG**GTGATGGA Loc:-997~-980 TATCGT**CGTG**TTGTGATT Loc:-1106~-1089 TTAAAC**CGTG**TGCGCTGC Loc:-55~-38	N/A	N/A

XTR	ENSXETG00000016375 TGTCTC**CGTG**TAATCGCG Loc:-1289~-1272 TAATCG**CGTG**CTGATAAC Loc:-1273~-1262	N/A	N/A

CIN	ENSCING00000014020 CAGATA**CGTG**ATCTTGGT Loc:--1977~-1960 TTACGA**CGTG**GACATTCC Loc:-1475~-1458 CGCCAT**CGTG**CGAAGGCA Loc:-150~-133	N/A	N/A

The locations of HRE motifs were detected within the HIF orthologous genes for 6 model species (the PSSM pattern of V$HIF_STKR_2005 and the core segment “CGTG” pattern were required to exactly match and shown in bold and underline). The regions were identified within 2,000 base pairs upstream of the orthologous gene set of KEGG: Orthology K05448 (VEGFA_B); only the first 3 detected HREs are shown here. The Ensembl gene IDs are shown on the top of each identified HRE motif. Only 2 HRE motifs were found in XTR within the assigned promoter region. HSA, *H. sapiens*; MMU, *M. musculus*; GGA, *G. gallus*; DRE, *D. rerio*; XTR, *X. tropicalis*; CIN, *C. intestinalis*

### Paralogous and orthologous gene relationships between 2 species

The proposed methodology provides a new paradigm for investigating functional diversification through mapped biological pathways. A gene exhibits different biological functions when it participates in different functional pathways. In the example in Figure 1, each node in the HIF pathway denotes the proportional number of paralogous genes and the orthologous property between the reference species (HSA) and the query species (DRE). Black background squares indicate that DRE might possess greater flexibility and the possibility of gene substitution than HSA. Gray background squares indicate equivalent numbers of paralogous genes, while white squares indicate that DRE does not possess any correlated orthologous gene in this pathway. When a specific biological pathway is selected for a particular organism, a mapped functional pathway based on the orthologous relationship between the query and reference species is constructed; each node in the pathway is displayed with different shades of gray according to the ParaRate values. Hence, the constructed pathways enhance the visualization effects of the homology and functional conservation between 2 species. Although the biological pathways predicted *in silico* under hypothetical assumptions may not be accurate, the mapped functional pathways based on cross-species comparison indeed provide clues for explaining functional conservation and alternative solutions. At present, the functional pathway mapping analysis only focuses on 6 selected model species; more model species will be gradually included for comprehensive analyses. In summary, the proposed methods can systematically discover orthology at the level of biological pathways and not merely with individual genes. In addition to genes, functional pathways are also conserved in general evolutionary processes. Analyzing the conserved pathways through cross-species comparison may help biologists to discover and distinguish the diversity of functional pathways. The comparison results may serve as a powerful tool for understanding physiological mechanisms in order to suggest better model species for subsequent *in vitro* or *in vivo* experimental designs.

### Conserved transcription factor analysis for orthologous and paralogous genes

To further verify the functional conservation of retrieved homologous genes from various species, conserved TFBSs in the promoter regions of homologous gene sets were identified. The TRANSFAC library version 10.3 was adopted for ordinary TFBS analysis. More than 800 TF-binding specificities (585 for vertebrate species) are summarized as PSSMs for TFBS findings. Users can select corresponding TF-binding motif patterns or define customized PSSM patterns to verify common transcription elements. Two examples of HIF orthologous and paralogous gene sets compared to randomly selected gene sets are shown in Tables 3 and 4. For comparison, 3 known HIF-related TFBSs from TRANSFAC and 1 customized V$HIF_STKE_2005 pattern were assigned to identify the conservation status of the target gene set. In the upper half of Table 3, orthologous genes were obtained from 6 model species at the “VEGF” node within the HIF pathway (KEGG: Orthology K05448). Each cell in the table shows the numbers of TFBSs retrieved by setting 3 different cut-off values: 0.85, 0.80, and 0.75. For example, the V$AHRHIF_Q6 motif for HSA is 5/5/29, indicating that the numbers of V$AHRHIF_Q6 motifs identified within the promoter region of the VEGF gene of HSA are 5, 5, and 29 by setting cut-off values of 0.85, 0.80, and 0.75, respectively. In lower half of Table 3, a gene set without an orthologous relationship was randomly selected from 6 species. The rightmost columns of Table 3 reveal that when cut-off values are low, the numbers of identified HIF TFBSs and conserved species both increase. It should be noted that the orthologous gene set possessed a greater number of conserved transcription elements than a randomly selected gene set, even when the cut-off value was as low as 0.75. In Table 4, the functional conservation of a paralogous gene set was also evaluated by comparing the number of conserved TF-binding motifs for both the paralogous gene set and a randomly selected gene set from an identical species. The paralogous gene set was obtained from the VEGF node within the HIF pathway of DRE, and the assigned PSSM patterns were the same as those of the previous example in Table 3. It is also noticeable that the number of identified conserved HIF TFBSs from the paralogous gene set is also greater than that of the randomly selected gene set. The results of both illustrated examples are consistent with the assumptions that homologous genes possess greater functional similarities. The identification of conserved TFBSs regarding a specific biological pathway can be further applied for determining functional conservation among various species.

**Table 3:** The number of identified HIF-related TFBSs within an orthologous gene set and a randomly selected gene set

Comparison for HIF orthologous gene set							
TF ID	HSA	MMU	GGA	CIN	DRE	XTR	# of species possessing the identical TFBS (0.85/0.80/0.75)

V$HIF_STKE_2005	0/0/2	1/1/3	1/1/3	0/0/1	0/0/2	0/0/0	2/2/5

V$HIF1_Q5	1/3/7	2/2/12	1/3/10	2/2/6	1/1/4	0/1/4	5/6/6

V$HIF1_Q3	1/3/10	2/2/12	1/4/9	2/3/7	1/1/4	0/0/11	5/5/6

V$AHRHIF_Q6	5/5/29	3/3/24	5/5/22	6/6/23	3/3/21	2/2/18	6/6/6

Total identified motifs	7/11/48	8/8/51	8/13/44	8/11/37	5/5/31	2/3/33	-

Comparison for a randomly selected gene set							

V$HIF_STKE_2005	0/0/1	0/0/0	1/1/4	0/1/2	1/1/2	0/0/0	2/3/4

V$HIF1_Q5	0/0/3	0/0/3	4/5/6	2/2/8	2/3/13	0/1/4	3/4/6

V$HIF1_Q3	0/0/4	0/0/1	4/4/6	2/3/14	2/2/7	0/0/11	3/3/6

V$AHRHIF_Q6	1/1/21	0/0/21	5/5/20	5/5/16	7/7/27	2/2/18	5/5/6

Total identified motifs	1/1/29	0/0/25	14/15/36	9/11/40	12/13/49	2/3/33	-

Comparison of TFBS conservation of the HIF orthologous gene set and a randomly selected gene set with 3 different cut-off values. (Upper half) The orthologous genes were obtained from 6 model species at the “VEGF” node within the HIF pathway (KEGG: Orthology K05448). These genes include ENSG00000119630 (HSA), ENSMUSG00000004791 (MMU), ENSGALG00000010290 (GGA), ENSCING00000014020 (CIN), ENSDARG00000034700 (DRE), and ENSXETG00000016375 (XTR). Three HIF-related TFBSs selected from the TRANSFAC library (V$HIF1_Q3, V$HIF1_Q5, and V$AHRHIF1_Q6) and 1 customized HRE motif (V$HIF_STKE_2005) were employed for scanning through all of the selected genes. Each cell in the table represents the numbers of retrieved TFBSs using 3 different cut-off values of 0.85, 0.80, and 0.75. For example, the V$AHRHIF_Q6 for HSA is 5/5/29, which represents the total number of V$AHRHIF_Q6 motifs that can be identified within the promoter region of the *Homo sapiens* VEGF gene by setting cut-off values at 0.85, 0.80, and 0.75. (Lower half) A set of genes without an orthologous relationship, randomly selected from 6 species. These genes include ENSG00000001460 (HSA), ENSMUSG00000073489 (MMU), ENSGALG00000000002 (GGA), ENSCING00000002344 (CIN), ENSDARG00000000086 (DRE), and ENSXETG00000016375 (XTR). Regarding the HIF function, most of the orthologous genes from various species indeed contain HIF TFBSs; however, they are sparsely distributed within the randomly selected genes. The rightmost columns also reflect the fact that the total number of species possessing identical HIF TFBSs is relatively higher within the orthologous gene set, since they are expected to retain HIF functional characteristics through the evolutionary process of speciation. HSA, *H. sapiens*; MMU, *M. musculus*; GGA, *G. gallus*; DRE, *D. rerio*; XTR, *X. tropicalis*; CIN, *C. intestinalis*

**Table 4:** The number of identified HIF-related TFBSs within a paralogous gene set and a randomly selected gene set of zebrafish species

Comparison for the paralogous genes				
TF ID	KEGG dre30682	KEGG dre403049	KEGG dre678512	# of gene possessing the identical TFBS (0.85/0.80/0.75)

V$HIF_STKE_2005	0/0/2	0/0/0	0/0/2	0/0/2

V$HIF1_Q5	1/1/4	0/0/3	2/3/3	2/2/3

V$HIF1_Q3	1/1/4	0/0/2	2/3/5	2/2/3

V$AHRHIF_Q6	3/3/21	0/0/15	4/4/30	2/2/3

Total identified motifs	5/5/31	0/0/20	8/10/40	-

Comparison for randomly selected genes				

	G1	G2	G3	

V$HIF_STKE_2005	0/0/1	0/0/0	0/0/0	0/0/1

V$HIF1_Q5	1/1/5	0/0/0	0/0/4	1/1/2

V$HIF1_Q3	1/1/9	0/0/1	0/0/3	1/1/3

V$AHRHIF_Q6	3/3/25	0/0/13	1/1/21	2/2/3

Total identified motifs	5/5/40	0/0/14	1/1/28	-

Comparison of TFBS conservation of the HIF paralogous gene set and a randomly selected gene set from the zebrafish (DTR) with 3 different cut-off values. (Upper half) The paralogous genes were obtained from the “VEGF” node within the HIF pathway. These genes include ENSDARG00000034700 (KEGG: dre30682), ENSDARG00000069640 (KEGG: dre403049), and ENSDARG00000056624 (KEGG: dre678512). The selected HIF TFBS patterns are the same as those in Table 3. Each cell in the table represents the number of retrieved TFBSs using 3 different cut-off values of 0.85, 0.80, and 0.75. For example, the V$AHRHIF_Q6 for the first DTR gene (ENSDARG00000034700) is 3/3/21 which represents the total number of V$AHRHIF_Q6 motifs can be identified within the promoter region (2,000 base pairs) of the *Danio rerio* VEGF gene by setting cut-off values of 0.85, 0.80, and 0.75. (Lower half) A set of genes without a paralogous relationship, randomly selected from zebrafish. These genes include ENSDARG00000040535 (G1), ENSDARG00000068465 (G2), and ENSDARG00000010610 (G3). Regarding the HIF function, most of the paralogous genes from a given node in the HIF functional pathway contain the corresponding HIF TFBSs but appeared less frequently in the randomly selected genes. The rightmost columns also reflect the tendency that the total number of gene possessing identical HIF TFBSs is relatively higher within the paralogous gene set since they might retain HIF functional characteristics through the evolutionary process of gene duplication.

## Discussion

To understand the mechanism behind hypoxia signal transduction, the HIF pathway from KEGG was initially extracted and analyzed. By calculating a newly defined ParaRate, the proposed methodology can discover alternative solutions that may possess high possibilities of replacing original genes at each node within a pathway without losing biological function. According to the obtained OrthRate percentages, the system derives similar gene clusters through cross-species comparison and predicts the functional pathway for the query species. Comparing paralogous genes obtained from a set of target genes facilitates the discovery of probable substitute genes with respect to a specific biological pathway. Accordingly, it is possible to construct a novel functional subpathway with respect to these identified alternative selections. When studying a functional pathway, combinatorial regulation by transcription factors and genes need to be analyzed and discussed simultaneously based on an assumption that the genes within the same pathway can be controlled by common regulators. Hence, a set of genes from the mapped functional pathway can be verified by focusing on the identification of common transcription factor-binding motifs.

## Conclusion

We propose a strategy for mapping functional pathways among various species that allows the retrieved orthologous and paralogous genes to be further verified by identifying common TF-binding motifs. Six model species including *H. sapiens* (HSA), *M. musculus* (MMU), *G. gallus* (GGA), *D. rerio* (DRE), *X. tropicalis* (XTR), and *C. intestinalis* (CIN) were used for cross-species comparison. The novel terms OrthRate and ParaRate of the mapped functional pathways were defined to quantitatively indicate the conservation and flexibility of homologous pathways. These calculated values can be applied to enhance the alternative selection of functional genes. To verify the gene replaceability within the inferred pathway through *in silico* analysis, a conventional strategy was executed by searching for TFBSs on all retrieved homologous genes. Here, the TRANSFAC database was adopted for discovering all conserved transcription elements based on the summarized PSSMs. From the cross-species analysis of mapped HIF pathways, 4 species (HSA, MMU, GGA, and DRE) possess highly similar mechanisms for maintaining HIF function compared to XTR and CIN. Furthermore, the corresponding TFBS analyses were evaluated with consistent performance.

## Methods

The initial pathway information was acquired from KEGG, a knowledge database for the systematic analysis of gene functions, to link genomic information with higher-order functional information [21,22]. Each map or pathway in KEGG was categorized into an existing taxonomy according to its function, and each pathway was supplemented with a set of orthologously grouped tables for cross-species information with respect to conserved pathways. The orthologous table summarizes functional correlations in the pathway, physical correlations in genomes, and evolutionary relationships among species. It provides useful information as a reference dataset for functional annotations. Using the HIF pathway as an example, if the user enters the keywords “hypoxia-inducible factor” into the main interface of KEGG (http://www.genome.jp/kegg/pathway.html), the system responds with only 1 entry: map05211. In addition to the searched pathways, users can adopt orthologous information from the KEGG and/or Ensembl databases. In particular, orthologous genes identified in KEGG are not only obtained by evaluating sequence similarity, but also by determining if all constituent members are verified within a functional group, such as a conserved subpathway or a molecular complex. The variations of derived datasets from these 2 resources provide hints for possible modifications of an accurate pathway.

### Quantitative measurement of functional pathways

To compare the expression level of orthologous gene clusters in pathways against others by utilizing a quantitative measure, it is necessary to represent those associated genes as mathematical objects and provide measurable indices for effective representation. Here, we define 2 types of homologous rate, OrthRate and ParaRate, which can quantitatively suggest alternative functional genes. OrthRate is defined as the total number of corresponding orthologous genes within a specified pathway from the query species divided by the total number of associated genes within the identical pathway from the reference species. OrthRate indicates the proportional percentage of corresponding genes within cross-species biological pathways. ParaRate is similarly defined as the total number of corresponding paralogous genes within a specified pathway from the query species divided by the total number of associated genes within the identical pathway from the reference species. ParaRate is considered as a replacement ratio of duplicated genes with respect to a specified biological function. According to statistical results, the system can be expected to retrieve possible missing subpathways within an individual species and predict extra direct and/or indirect pathways within each species. To demonstrate the functional conservation and alternative selection of genes among various species, 6 remote model species including *C. intestinalis* (CIN), *X. tropicalis* (XTR), *G. gallus* (GGA), *M. musculus* (MMU), *D. rerio* (DRE), and *H. sapiens* (HSA) were initially considered for orthologous analysis in this study. Users are required to define both the query and reference species in advance. The quantitative measurement of functional pathways can then be obtained by taking the total number of homologous genes within the pathway of the reference species as the denominator and the total number of homologous genes of the query species as the numerator. In this study, both the numbers of orthologous and paralogous genes were obtained from the KEGG database.

### Identification of HRE motifs

To demonstrate the validity of *in silico* pathways in the biological sense, the existence of TFBSs within HIF target genes were considered as the verification criterion. HIFs bind target genes at the functional hypoxia response elements (HREs). An overview of the known target genes of HIF reveals that the length of a HRE is nearly 18 base pairs. The mandatory core HRE sequence is “CGTG”—the minimal DNA motif required for interaction with HIFs. The appearance frequency of HREs located within the flanking sequence is randomly distributed as reported previously [6]. To perform alignment and identify whether the HREs are located within the paralogous genes, we created a position-specific scoring matrix (PSSM) to extract all HRE candidates from retrieved HIF target genes. The PSSM matching mechanism scans through a DNA sequence with a fixed length and identifies the most probable motifs according to the calculated scores and sum of position-specific scores for each symbol in the verified substring [23,24]. The score value of each substring was obtained by summarizing the corresponding scores of PSSM to the *j^t^*^h^ substring *S_j_* within the DNA sequence, and the value was calculated as , where *i* represents the position in the substring, *S_i_* is the nucleotide symbol at position *i* in the substring, and *p_k_*_,_*_i_* is the score values in row *k*, column *i* of the PSSM matrix of a specific TF-binding pattern *P*. The PSSM profile of the HRE patterns for HIF target genes was generated according to the published paper by R. H. Wenger *et al*. [6] and was called the pattern matrix of V$HIF_STKE_2005. The HRE motif searching mechanism allowed us to verify the functional conservation of HIF responses within the retrieved homologous gene set.

### Identification of common transcription factors

The identification of common TFBSs is an *in silico* analysis for ensuring that the target genes possess identical biological functions [25] under the assumption that a homologous gene set within the same pathway can be controlled by common regulators. Therefore, we employed the TRANSFAC database for identifying the common TFBSs of a specified HIF target gene set. The TRANSFAC library version 10.3 is a comprehensive set of more than 800 TF-binding specificities (585 for vertebrate species), which was adopted and summarized as PSSMs to search transcription elements. The default PSSM cut-off value of each TF-binding pattern is set as 0.85 for examining all substrings from the selected candidate genes; the common TFBSs identified for all selected homologous genes are displayed in priority order according to the appearance pervasiveness and frequency among the defined gene set. In the developed system for conserved TFBS analysis, users are allowed to select limited TFBSs through keyword filtering functions, and the selected PSSM can be from either the TRANSFAC library or user defined matrices. In particular, 3 HIF-related PSSM matrices, including V$HIF_Q3, V$HIF_Q5, and V$AHRHIF_Q6, were assigned along with the customized factor V$HIF_STKE_2005 for analyzing the orthologous and paralogous gene sets within the HIF pathway.

## Competing interests

The authors declare that they have no competing interests.

## Author contributions

CSC developed the systems and performed evaluations. TWP conceived the study and drafted the manuscript. MDC validated promoter analysis and proofread the manuscript; CHH, WST, HTC, and CCC participated in the system design and evaluation.
